# Enhanced third-harmonic generation by manipulating the twist angle of bilayer graphene

**DOI:** 10.1038/s41377-020-00459-5

**Published:** 2021-01-21

**Authors:** Seongju Ha, Nam Hun Park, Hyeonkyeong Kim, Jiseon Shin, Jungseok Choi, Sungmin Park, Ji-Yun Moon, Kwanbyung Chae, Jeil Jung, Jae-Hyun Lee, Youngdong Yoo, Ji-Yong Park, Kwang Jun Ahn, Dong-Il Yeom

**Affiliations:** 1 Department of Energy Systems Research, Ajou University, 206 Worldcup-ro, Yeongtong-gu, Suwon, 16499 Republic of Korea; 2 Korea Research Institute of Standards and Science, Daejeon, 34113 Republic of Korea; 3 Department of Physics, University of Seoul, 163 Siripdaero, Dongdaemun-gu, Seoul, 02504 Republic of Korea; 4 Department of Smart Cities, University of Seoul, 163 Siripdaero, Dongdaemun-gu, Seoul, 02504 Republic of Korea; 5 Department of Materials Science and Engineering, Ajou University, 206 Worldcup-ro, Yeongtong-gu, Suwon, 16499 Republic of Korea; 6 Department of Chemistry, Ajou University, 206 Worldcup-ro, Yeongtong-gu, Suwon, 16499 Republic of Korea; 7 Department of Physics, Ajou University, 206 Worldcup-ro, Yeongtong-gu, Suwon, 16499 Republic of Korea

**Keywords:** Nonlinear optics, Optical properties and devices, Nonlinear optics

## Abstract

Twisted bilayer graphene (tBLG) has received substantial attention in various research fields due to its unconventional physical properties originating from Moiré superlattices. The electronic band structure in tBLG modified by interlayer interactions enables the emergence of low-energy van Hove singularities in the density of states, allowing the observation of intriguing features such as increased optical conductivity and photocurrent at visible or near-infrared wavelengths. Here, we show that the third-order optical nonlinearity can be considerably modified depending on the stacking angle in tBLG. The third-harmonic generation (THG) efficiency is found to significantly increase when the energy gap at the van Hove singularity matches the three-photon resonance of incident light. Further study on electrically tuneable optical nonlinearity reveals that the gate-controlled THG enhancement varies with the twist angle in tBLG, resulting in a THG enhanced up to 60 times compared to neutral monolayer graphene. Our results prove that the twist angle opens up a new way to control and increase the optical nonlinearity of tBLG, suggesting rotation-induced tuneable nonlinear optics in stacked two-dimensional material systems.

## Nonlinear optics: When graphene ‘does the twist’

Modifying the angle between the two layers of twisted bilayer graphene can considerably improve how photons interact with it to generate higher-energy photons. Dong-Il Yeom of Ajou University and colleagues in Korea shined laser light onto twisted bilayer graphene stacked at various angles, and measured the outcoming optical signals. Twisted bilayer graphene is made by stacking two monolayer graphenes at an angle. The interlayer interaction in this material leads to intriguing properties. The scientists found that changing the angle could enhance a nonlinear optical property, called third-harmonic generation, by 60 times compared to monolayer graphene. The team then conducted Raman measurements and modeling calculations to understand how this process occurs. They say their findings could pave the way towards novel designs for enhancing optical nonlinearity in two-dimensional stacked materials.

## Introduction

Nonlinear optical processes occur efficiently when highly intense light interacts with a material^1,2^. Strong light-material interactions play an essential role in enhancing the nonlinear polarization response of a material, thus improving the efficiency of the generated nonlinear signal. Two-dimensional (2D) layered materials, such as graphene and transition metal dichalcogenides, have recently received increasing attention as promising materials for nonlinear optical applications because of their unique electric and optical features^3,4^. Graphene, the first experimentally discovered 2D material^5^, consists of carbon atoms forming a hexagonal periodic single layer and hence is centrosymmetric. As second-order nonlinearity is not allowed in graphene possessing inversion symmetry, the third-order nonlinear optical process becomes the primary nonlinear response. It is well known that graphene has a very large third-order optical nonlinearity over a broad bandwidth due to its strong optical coupling and linear energy dispersion of Dirac fermions^6^. Numerous studies have been actively conducted on third-order optical nonlinearity in graphene, such as on third-harmonic generation (THG)^7–12^, four-wave mixing^11,13,14^, optical bistability^14^, and nonlinear saturable absorption for laser mode locking^15–17^.

Twisted bilayer graphene (tBLG) consists of a pair of graphene monolayers stacked with misorientation in the crystal axes, forming Moiré superlattices of carbon atoms^18^. The additional static potential due to the interlayer interaction in tBLG notably reconstructs the electronic band structure of the base material, allowing us to observe very intriguing physical properties, including superconductivity^19,20^, Mott insulators^20,21^, magnetism^22^, and Moiré excitons^23^, which are not visible in monolayer graphene (MLG). In tBLG, a divergence in the electronic density of state, called a van Hove singularity (VHS), shows a dependence on the twist angle between layers^24^. The rotation-induced VHS can appear at a lower bandgap energy (under 3.9 eV), which enables the observation of interesting features such as resonant optical conductivity^25–27^, increased photoexcited currents^28^, and strongly enhanced Raman G-band signatures at visible or near-infrared wavelengths^29–31^.

Here, we report, for the first time to our knowledge, that the third-order optical nonlinearity can be considerably modified depending on the stacking angle in tBLG. We measured the nonlinear optical response of various tBLG samples stacked with different twist angles and observed that there was a strong enhancement of the THG in tBLG at a specific twist angle. Through Raman measurement and continuum model calculation, we discovered that the enhanced THG occurred when the energy gap of the VHS coincides with the three-photon resonance of the incident light. Furthermore, we examined THG characteristics by electrically controlling the third-order nonlinear optical susceptibility of tBLG through ion-gel gating. The enhancement factor of the THG signal due to electrical gating varies depending on the twist angle, where the maximum value of the THG intensity in tBLG is approximately 60 times that in neutral MLG. Our results provide a basic understanding of third-order nonlinear optical responses having a strong relationship with the twist angle in tBLG, which paves a novel way for designing and enhancing the optical nonlinearity in 2D stacked materials.

## Results

### Sample characterization

**Fig. 1:**
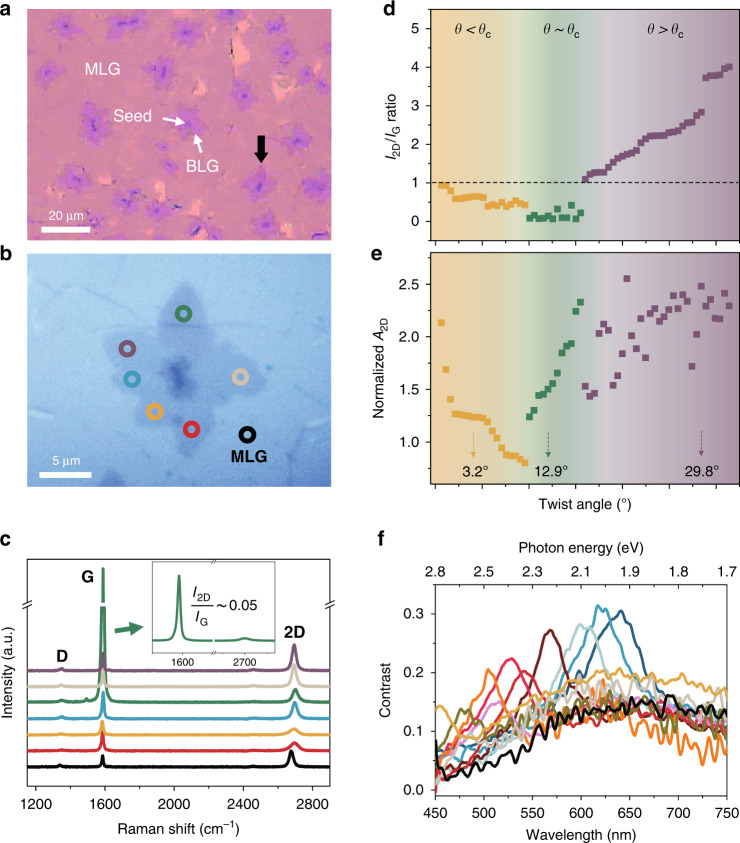
Optical and Raman characteristics of CVD-grown tBLG. **a** Microscope image of CVD-grown graphene transferred onto a 300 nm SiO_2_/Si substrate. MLG covers most of the silica area with locally distributed star-shaped tBLG centred at the seeds (the darkest spots in the image). **b** Enlarged microscope image of the tBLG indicated by the black downward arrow in (**a**). Colour circles represent individual tBLG regions according to the twist angles. The black-circled region represents MLG. **c** Raman spectra of tBLG marked with the same colour as the circles in (**b**). Inset: Raman spectrum showing the full G- and 2D-bands in the green-circled tBLG in (**b**), where Raman G-resonance occurs from the interaction with the 532 nm excitation laser, indicating that the twist angle should be 12 ± 1° (refs. ^29,33^) (the critical angle, *θ*_*c*_, regime). Various Raman signals measured in 63 tBLG regions (**d** 2D/G peak ratio, and **e** 2D-band area normalized by that of MLG) were used to classify the twist angle of tBLG. For clarity, tBLG regions with *θ* < *θ*_*c*_, *θ* ∼ *θ*_*c*_, and *θ* > *θ*_*c*_ are represented by orange-, green- and purple-coloured sections, respectively. **f** Linear absorption contrast spectra in tBLG on a 100 nm SiO_2_/Si substrate

Graphene samples for characterization of third-order optical nonlinearity were synthesized by copper-catalysed chemical vapor deposition (CVD). Figure 1a shows an optical microscope image of our sample transferred onto a 300 nm-thick SiO_2_/Si substrate via the PMMA-assisted wet transfer method. In a wide area of monolithic MLG, a considerable star-shaped overgrown region of graphene around a seed can be observed. Through AFM measurement, we confirmed that this overgrown area is bilayer graphene (BLG) with a height difference of 0.33 nm from MLG (see Supplementary Fig. S1).

The CVD-grown star-shaped graphene area consists of BLG exhibiting locally varying misorientation in the crystals between the top and bottom graphene layers^29,32^, which provides a suitable platform to investigate the twist angle-resolved THG in tBLG. We performed Raman spectroscopy on a star-shaped graphene area (for example, at each location marked with coloured circles in Fig. 1b) with a 532 nm excitation laser. A high magnification objective lens (100×, 0.85 NA) was used to focus the light to a small spot size of ~1 μm, and graphene was irradiated by the laser for 5 s with an average incident power of 1 mW to prevent thermal damage. Figure 1c shows the measured Raman spectra, where the colour of the line indicates each measured BLG spot in Fig. 1b. The Raman spectra show different features (such as peak intensity, full-width at half-maximum (FWHM), and Raman shift of the G- and 2D-bands) arising from the diverse photon-phonon interactions, indicating that each star-shaped graphene area consists of tBLG with various twist angles^29,32^ and a seed at the centre.

One of the interesting features of the Raman response is that an enhanced G-band signal could appear when the photon energy of the excitation laser corresponds to the energy gap of the VHS in tBLG^29,30,32,33^. The green line in Fig. 1c shows the resonantly enhanced G peak for the 532 nm excitation laser used in our Raman measurement, accompanying a very small 2D/G peak ratio of ~0.05, as shown in the inset. The matched energy gap of the VHS in tBLG is approximately 2.33 eV, where the twist angle is estimated to be 12 ± 1° (refs. ^29,33^). The twist angle at the excitation laser resonance is called the critical angle, *θ*_*c*_. In tBLG with twist angle smaller than *θ*_*c*_ (*θ* < *θ*_*c*_), the photon energy of the excitation laser is larger than the energy gap of the VHS, and Raman signals occur in more complex forms because of the expanded Moiré superlattices and the increased chances of interlayer interaction^29,30^. In this region, the 2D/G peak ratio is less than unity with broadened FWHM of the 2D-band (blue-, yellow- and red-coloured spectra in Fig. 1c). On the other hand, for tBLG with a twist angle of *θ* > *θ*_*c*_, the energy gap of the VHS is larger than the photon energy of the excitation laser. Thus, the photoexcited electrons hardly interact with the newly generated VHS, resulting in Raman signals similar to those of MLG^29,30^, featuring a 2D/G peak ratio above unity (grey- and purple-coloured spectra in Fig. 1c).

Here, we analysed 63 tBLG regions in total via Raman spectroscopy using a 532 nm excitation laser, where the tBLG regions were classified into three groups: *θ* < *θ*_*c*_, *θ* ∼ *θ*_*c*_, and *θ* > *θ*_*c*_. To select tBLG regions belonging to the *θ* < *θ*_*c*_ and *θ* > *θ*_*c*_ groups, the abovementioned features of the 2D/G peak ratio and FWHM of the 2D-band were primarily considered. On the other hand, we distinguish tBLG belonging to the *θ* ∼ *θ*_*c*_ group based on the 2D/G peak ratio and G-band area normalized by that of MLG in the Raman signal. In Fig. 1d, e, we compare the quantitative differences in the 2D/G peak ratio and normalized area of the 2D peak according to the tBLG regions categorized by twist angle; these trends are in good agreement with the previous reports^29,30,34^. The twist angles of several points, estimated from the measurement of the electron beam diffraction pattern described below, are shown together in Fig. 1e. We measured the linear absorption contrast spectra in the tBLG, defined as *C*(*λ*) = (*R*_0_(*λ*) − *R*(*λ*))/*R*_0_(*λ*), where *R*_0_(*λ*) and *R*(*λ*) are the reflection spectra from the MLG and tBLG, respectively^25^. *C*(*λ*) obtained at various tBLG positions is shown in Fig. 1f. Each curve shows the distinct absorption band characteristics associated with the low-energy VHS in tBLG. In addition, we measured the electron beam diffraction pattern by using transmission electron microscopy (TEM) to confirm the twist angle in tBLG. The results of TEM measurements at several positions in tBLG are shown in Supplementary Fig. S2, which are consistent with our expectations based on the Raman and contrast spectral measurements.

### Third-harmonic generation in tBLG

**Fig. 2:**
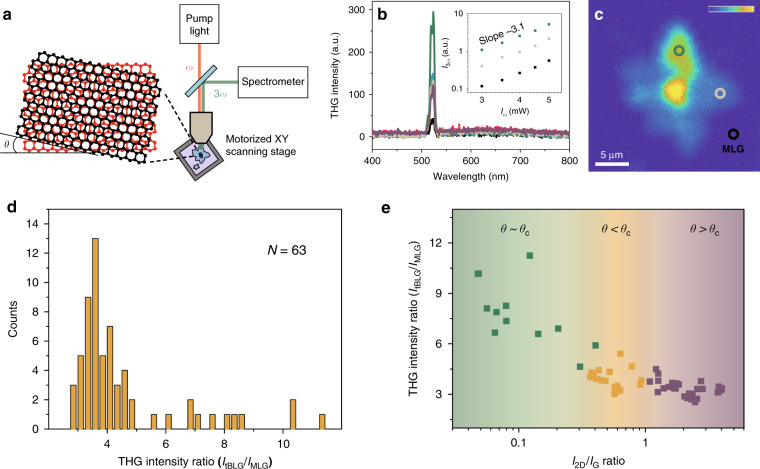
***θ***-dependent THG responses in tBLG. **a** Schematic of our lab-made reflection-type measurement system for nonlinear optical responses in tBLG. **b** Spectra of nonlinear optical signals from MLG and tBLG over visible wavelengths. The dominant spectra show the typical THG signals centred at 520 nm. Inset: output power dependence of THG signals on pump power. The slopes are near 3, indicating intrinsic THG characteristics. These data were measured at the MLG (black circle) and tBLG (grey and green circle) positions in (**c**). **c** THG image of the graphene region in Fig. 1b, scanned by motorized XY stages with a 0.3 μm step resolution over 30 μm × 30 μm. **d** Histogram of the THG intensity ratio for 63 tBLG regions and MLG. **e** Tendency of the THG intensity arranged with respect to the 2D/G peak ratio

To investigate the nonlinear optical signals from tBLG, a lab-built reflection-type measurement system was adopted with an ultrafast mode-locked fibre laser providing reliable femtosecond pulses centred at 1560 nm (Fig. 2a, see ‘Materials and methods’ for details). Figure 2b presents the spectral characteristics of the nonlinear optical signals generated from MLG and tBLG between 400 nm and 800 nm. The colours of each spectrum correspond to the coloured circles marked on MLG and tBLG in Fig. 1b. The dominant spectra show typical THG signals centred at 520 nm in accordance with a third of the wavelength of our fs pulse laser (also see the THG intensity that scales with the cube of the incident laser power in the inset of Fig. 2b). In addition, we observed upconverted nonlinear ultrafast photoluminescence^35–38^ broadly distributed over 530–780 nm with very weak intensity, and no SHG signal was detected within the sensitivity range of our measurement.

Most regions of the tBLG area show THG intensities from 3.5 to 4 times that of MLG, which is consistent with the general feature of the THG signal being proportional to the square of the layer number in graphene^7–10^. However, the tBLG area with a specific twist angle shows a much higher THG intensity. For example, the nonlinear signal at the green circle in Fig. 1b is approximately ten times that of MLG. Spatial mapping of the THG intensity in Fig. 2c distinguishes the areas of monolayer, bilayer, and seed graphene. In particular, tBLG exhibiting an enhanced THG signal is clearly visible in a specific region. To investigate the features of the THG in tBLG with various twist angles, we experimentally examined the nonlinear optical signals for 63 regions of tBLG (see examples of several tBLG samples in Supplementary Fig. S3), and Fig. 2d summarizes the results as a histogram of the THG intensity ratio. More than 80% of tBLG regions are included in a Gaussian-like distribution with a centre value of 3.5, showing an aspect of BLG. Meanwhile, the cases showing an enhanced THG ratio of > 6 occupy a certain weight (~17%) in the histogram. We plot the THG intensity of the 63 tBLG regions as a function of the 2D/G peak ratio and present the results in Fig. 2e. The THG intensity tends to increase as the 2D/G peak ratio decreases in all groups. In particular, tBLG in the *θ* ∼ *θ*_*c*_ group shows more enhanced THG intensity as the 2D/G peak ratio decreases. Since the 2D/G peak ratio generally decreases when approaching the critical angle region for the *θ* > *θ*_*c*_ and *θ* < *θ*_*c*_ groups (see Fig. 1d), it is expected that the trend appearing in Fig. 2e results from the resonance effect around the rotation-induced VHS.

**Fig. 3:**
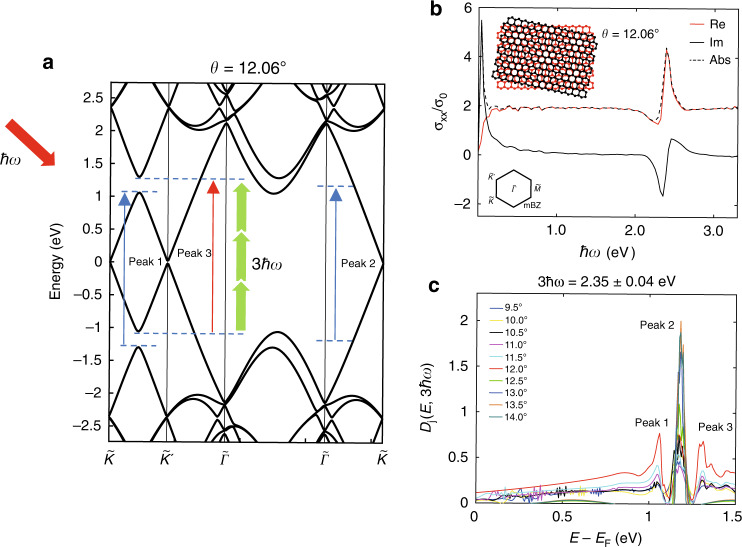
Enhanced THG in tBLG via resonantly increased linear optical conductivity. **a** Schematic of the energy band diagram of tBLG when the energy gap of the VHS matches the three-photon energy of the incident light. **b** Resonantly increased linear optical conductivity in tBLG with a twist angle of 12.06° calculated by the continuum model method. Inset: schematic of Moiré superlattices formed at the interface of tBLG with a twist angle of 12.06°. **c** Joint density of states of tBLG for different twist angles between 9.5° and 14°. The three peaks are denoted by the three arrows in (**a**)

The enhanced THG intensities in the tBLG are associated with contributions of increased interband transitions. In tBLG, the singularities in the DOS are formed at a specific frequency determined by the twist angle, which evolves almost linearly with the twist angle as $E_{VHS} \approx \pm \hbar v_FK_\theta$, where $\hbar v_F$ gives the slope of the Dirac cone, *K* is the momentum space Dirac cone position of graphene with respect to the Γ point at the origin, and *θ* is the twist angle. The enhancement in the density of states that accompanies this singularity causes a resonant increase in the linear optical conductivity, as noted in Fig. 3a. Thus, enhanced nonlinear optical susceptibility can also be expected when these newly generated real atomic states coincide with the photon energy of the incident harmonic waves^1,2^. Our experiment shows higher THG intensities in the *θ* ∼ *θ*_*c*_ group, where the energy gap (2.33 eV) of the induced VHS nearly matches the three-photon energy of the fs pump pulses at 1560 nm, as depicted in Fig. 3a. The enhanced THG in tBLG is expected to be attributed to the strongly resonant three-photon transition created in VHS states. The resonantly increased linear optical conductivity in tBLG calculated using the continuum model (see Materials and methods) is shown in Fig. 3b. We set the twist angle *θ* = 12.06°, with the energy gap of the VHS corresponding to the three-photon energy of the 1560 nm wavelength, as in our cases. The calculated result shows a nearly constant value of twice *σ*_0_ for all of the high energy regime except for the peak value of 4.82*σ*_0_ at an energy of ~2.35 eV, where *σ*_0_ is the universal conductivity $\sigma _0 = e^2/4\hbar$. We present the joint DOS of tBLG for a particular transition energy of 2.35 ± 0.04 eV at different twist angles in Fig. 3c. There are three prominent peaks that correspond to each of the three arrows in Fig. 3a. Peak 2, which is attributed to the possible transitions between $\tilde K^{\prime}$ and ${\tilde{\mathrm \Gamma }}$, occurs at half of the transition energy (*E*−*E*_*f*_) ~ 1.17 eV regardless of the twist angle. Peaks 1 and 3, which are the manifestations of the transitions at the $\tilde M$ point, stand out when the twist angle is 12.06°. For undoped graphene, the third-order nonlinear optical conductivity $\sigma ^{\left( 3 \right);xxxx}(\omega _1,\omega _2,\omega _3) \propto \sigma _0$ is proportional to the linear optical conductivity^6,13^, and likewise, considering that the low-frequency third-order susceptibility^1^ $\chi ^{( 3);xxxx}(\omega _0,\omega _1,\omega _2,\omega _3) \propto \mathop {\prod }\nolimits_{i = 0}^3 \chi ^{(1);xx}(\omega _i)$ is proportional to the linear susceptibilities in a classical atomic model, it can be argued that the joint DOS that accounts for the availability of initial and final excitation states in tBLG already gives an account of the possible enhancements in the nonlinear photon-electron coupling. Because the position of the VHS in tBLG that enhances the linear optical absorption can be tuned with the twist angle, it is expected that the nonlinear optical properties can also be actively controlled by engineering the twist angle in tBLG, as evidenced in our experimental observations.

### Electrical tuning of the third-order susceptibility in tBLG

**Fig. 4:**
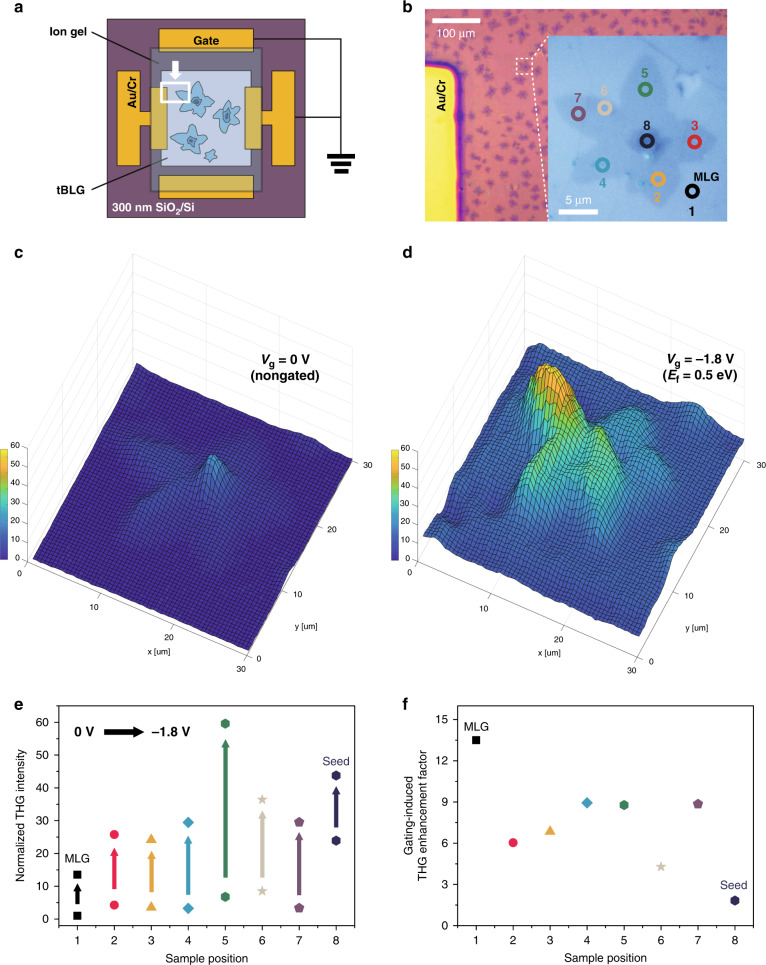
Third-order optical nonlinearity in tBLG electrically tuned by ion-gel-based top gating. **a** Schematic diagram of the ion-gel-coated top-gating graphene device. **b** Microscope image of the graphene area corresponding to the white-coloured rectangle (highlighted with a white arrow) in (**a**). Inset: enlarged microscope image of a selected tBLG region consisting of various twist angles represented with different colour circles, whose Raman spectra are shown in Supplementary Fig. S4b. Measured THG images of the tBLG region (inset in **b**) at **c** *V*_*g*_ = 0 V and **d** *V*_*g*_ = –1.8 V, where the strongest THG occurs from grey-circled tBLG and green-circled tBLG, respectively. **e** THG intensities normalized by the THG intensity of MLG at *V*_*g*_ = 0 V for each graphene position before/after applying a *V*_*g*_ of –1.8 V. **f** Gating-induced THG enhancement factor; the rate of increased THG intensity at *V*_*g*_ = –1.8 V compared to the THG intensity at *V*_*g*_ = 0 V at each graphene position. The number indicates the sample location shown in the inset in (**b**)

As a next step, we examined THG characteristics in tBLG while adjusting the Fermi level by electrical gating. Figure 4a shows a schematic of our ion-gel-coated gate device, which provides a uniform and strong electric field over a large area of tBLG (for details of the device fabrication, see ‘Materials and methods’). The electrical transport properties of our device were investigated by measuring the source-drain current along with the gate voltage (*V*_*g*_), and we found that the graphene layer shows p-doped characteristics with positive Dirac voltages (Supplementary Fig. S4a). Microscope images of our device are displayed in Fig. 4b. Among the numerous star-shaped tBLG regions, we selected the tBLG area (inset of Fig. 4b) composed of the twist angles covering all three groups; red, yellow and blue circles in *θ* < *θ*_*c*_, purple circle in *θ* > *θ*_*c*_, and grey and green circles in *θ* ∼ *θ*_*c*_, where the grey circle region exhibits the highest peak in the G-band (Supplementary Fig. S4b). Supplementary Fig. S5 shows the representative results of THG intensity versus applied *V*_*g*_ from 2 V to –2 V in tBLG. Here, as *V*_*g*_ increases to a negative value (towards p-type doping), it was observed that the THG intensity in tBLG with several twist angles increases monotonically without irregular changes or distortions in the spectral profile (for example, see the inset of Supplementary Fig. S5). THG intensities were measured over the entire tBLG region shown in the Fig. 4b inset at *V*_*g*_ of 0 V and –1.8 V, and the results are compared in Fig. 4c, d. Here, the Fermi energy of graphene is estimated to be ~0.5 eV at an applied *V*_*g*_ of –1.8 V considering the nonlinear enhancement factor in MLG described below. The mapping of nonlinear optical signals clearly distinguishes the areas separated by mono-, bi-, and multilayer graphene. At *V*_*g*_ = 0 V, the graphene seed shows the highest THG intensity (~24 times that in MLG), while the tBLG shows different enhancement values ranging from 3.3 to 8.5. When a *V*_*g*_ of –1.8 V is applied to the device, the THG mapping shows a more prominent feature, as shown in Fig. 4d, where the specific tBLG region exhibits a higher THG intensity than the graphene seed. Figure 4e displays the quantitative comparison where the THG intensities of each area normalized by that of neutral MLG are shown at *V*_*g*_ = 0 V and –1.8 V. The near-critical angle tBLG regions (grey- and green-circled areas in Fig. 4b) show the most enhanced normalized intensities of 8.5 and 6.8, respectively, at *V*_*g*_ = 0 V. In contrast, normalized THG intensities with values of 3.3–4.3 appear in other tBLG regions at *V*_*g*_ = 0 V. On the other hand, at –1.8 V, the green-circled tBLG shows the highest THG intensity of 60, followed by the value of 43.8 from the graphene seed. The grey-circled tBLG exhibiting the largest G peak in the Raman signal shows a value of 36 (for THG spectra in green- and grey-circled tBLG, see Supplementary Fig. S6). Other tBLG regions present values between 24 and 29 in normalized THG intensity, as shown in the figure.

The gating-induced THG enhancement factors defined as $I_{THG}(V_g = -1.8\,{\mathrm{V}})/I_{THG}(V_g = 0\,{\mathrm{V}})$ are shown in Fig. 4f. MLG has a THG enhancement factor of ~13.5, where the estimated Fermi energy is approximately 0.5 eV considering the previous work^11^. THG enhancement factors from 4.3 to 8.9 were obtained in our tBLG sample, which are all smaller than that in MLG. We expect that this might result from the screening effect and layer-dependent carrier concentration in BLG under top gating^34,39,40^. Due to this interlayer screening effect in graphene, multilayer graphene (*n* ≥ 2) inevitably has a potential difference between the layers, resulting in an exponential decrease in the carrier concentration from the top layer to the bottom layer in ion-gel-based top-gating experiments. Therefore, when *V*_*g*_ is applied to the device, the induced charges are distributed in tBLG, exhibiting different doping levels for each layer^39^, which leads to a smaller THG enhancement in tBLG than in MLG. The screening effect is also responsible for the lowest THG enhancement factor (1.7) at the graphene seed.

## Discussion

The developed potential difference of Δ*V* between the top and bottom graphene under gating causes reconstruction of the electronic band structure of tBLG. For example, two Dirac cones in the top and bottom graphene layers are shifted by ±*e*Δ*V*/2 with separation of two saddle points by ±*e*Δ*K*_*gate*_/2 from the *M*-point in the Brillouin zone (BZ) (see Supplementary Fig. S7a, b), resulting in an electron-hole asymmetric band structure. Previous studies reported splitting and quenching of the Raman G-band intensity in tBLG with an asymmetrically reconstructed band structure^34,39^. Similarly, we expect that the strong coupling with three-photon resonance in the intrinsic tBLG might diminish under gating, which causes the smallest enhancement factor of 4.3 in resonant tBLG (sample position 6 in Fig. 4f). On the other hand, the green-circled tBLG having a VHS energy gap near the three-photon energy of the pump source shows a THG enhancement factor of ~9 (sample position 5 in Fig. 4f), resulting in the strongest value of 60 in the normalized THG intensity. Supplementary Fig. S7c–f conceptually depicts the bandgap feature of the tBLG having a slightly smaller (red line) or larger (blue line) VHS energy gap than the three-photon resonance. When a negative *V*_*g*_ biases this tBLG, asymmetric separation of the VHS in the BZ occurs as mentioned above. This shifts the resonance of the optical conductivity in the vicinity of the initial resonance^40^, as schematically expressed in Supplementary Fig. S7d, f, which is expected to provide the highest enhancement of the THG signal under electric gating in green-circled tBLG.

We note that the previously reported third-order susceptibilities of MLG show large discrepancies ranging from 10^−15^ to 10^−19^ m^2^ V^−2^ depending on the measured wavelength, sample quality, type of substrate, doping level, etc^3^. Thus, it is more instructive to estimate *χ*^(3)^ through a relative comparison with $\chi _{MLG}^{(3)}$ obtained under the same experimental conditions^41^. We calculated the relative ratio of *χ*^(3)^ by using the relation^41^ $\left| {\chi _{tBLG}^{(3)}} \right|/\left| {\chi _{MLG}^{(3)}} \right| = d_{MLG}/d_{MLG}\sqrt {I_{tBLG}/I_{MLG}}$ where *d*_*MLG*_ and *d*_*tBLG*_ are the thicknesses of MLG and tBLG, respectively, and *I*_*MLG*_ and *I*_*tBLG*_ are the THG intensities of neutral MLG and tBLG, respectively. Table 1 summarizes the results. The normalized *χ*^(3)^ of tBLG is in the range of 0.91 to 1.46 without an applied *V*_*g*_, which increases to the range of 2.54 to 3.86 at the applied *V*_*g*_ of –1.8 V.

**Table 1:** Relative third-order susceptibility of tBLG compared with the THG intensity of neutral MLG at various sample positions at *V*_*g*_ = 0 V (nongated) and –1.8 V

Sample position $${\raise0.5ex\hbox{$\scriptstyle {\chi _{tBLG}^{(3)}}$}\kern-0.1em/\kern-0.15em \lower0.25ex\hbox{$\scriptstyle {\chi _{MLG}^{(3)}}$}}$$	1 (MLG)	2	3	4	5	6	7
*V*_g_ = 0 V	1.0	1.03	0.94	0.91	1.30	1.46	0.91
*V*_g_ = –1.8 V	3.67	2.54	2.46	2.71	3.86	3.02	2.72

In summary, we have studied the distinguished features of third-harmonic waves generated in tBLG stacked with different twist angles. An enhanced third-order nonlinear optical response was observed in neutral tBLG when the energy gap of the VHS in tBLG matched the three-photon resonance of the incident light. It is worth reporting that similar resonant behaviour in second-order nonlinear optical properties has been recently reported in undoped tBLG^42,43^. Additionally, we examined THG characteristics in tBLG with electrical gating, where we revealed that there exists an interdependent relationship between the twist angle of tBLG and the gate voltage for the third-order nonlinear optical responses. It is expected that the study of optical nonlinearities over a broad spectral range will help with obtaining an additional understanding, such as of the enhanced nonlinearity at each position of the multiphoton resonances, as well as with estimation of the electronic temperature^12^ in tBLG. Our results provide important observations on the twist-angle-dependent optical nonlinearity in tBLG, which paves the way towards the design of 2D-stacked materials for applications in future nonlinear photonic and optoelectronic devices that take advantage of the enhanced optical nonlinearity.

## Materials and methods

### Measurement of nonlinear optical responses from tBLG

To generate the nonlinear optical responses in tBLG, we used a mode-locked fibre laser as pump light with a centre wavelength of 1560 nm, a pulse duration of ~100 fs, a repetition rate of 80 MHz and an average maximum output power of >350 mW (Toptica, Femtopro IR). The incident pump power was controlled by a combination of a half-wave plate and a cube polarization beam splitter. Pump light of 5 mW was typically used in our measurements to clearly observe the generated nonlinear optical signals from tBLG and avoid thermal damage to the sample. The pump light was transmitted through a 950 nm longpass dichroic mirror and focused on the sample by an objective lens (40×, 0.75 NA) with a <2 μm spot size. The nonlinear optical signals reflected by the substrate were collected and collimated by the objective lens in the opposite direction. Then, the 950 nm longpass dichroic mirror reflected the generated nonlinear optical signals to be observed by a highly sensitive spectrometer (Ocean Optics, QEpro). Data for THG images were measured and saved using a combination of motorized XY stages and the software provided by Ocean Optics. For the electrical gating experiment, we measured the source-drain currents and applied *V*_*g*_ between the gate electrode and drain electrode using a Keithley 2400 series. All our measurements were conducted at atmospheric pressure at room temperature.

### Fabrication of the electrical gating device

CVD-grown graphene of 5 mm × 7 mm was transferred to the centre of a 300 nm SiO_2_/Si substrate via a well-known PMMA-assisted wet transfer technique. We used a 20 mm × 20 mm chrome shadow mask patterned with four electrodes, two of which were for connection of the active channel area with a 5 mm length, and the others were for top gating with a 7 mm distance (yellow-coloured parts in Fig. 4a). Chrome (5 nm) and gold (45 nm) were deposited on the sample through the E-beam evaporation process. Next, a [TFSI]^−^-based ion gel was employed via the spin-coating method (2500 rpm, 45 s) over the whole sample surface with a thickness of 2–3 μm. Finally, we eliminated unnecessary parts of the ion gel and connected the aluminium wires to apply *V*_*g*_ and measure the electrical signals.

### Continuum model calculation

The numerical calculation of the band structure of a twisted bilayer graphene system generally faces the complexity of having to deal with a large number of atoms in a periodic supercell based on the conventional Bloch’s theorem for solids. We circumvented such a heavy and time-consuming burden of a direct real-space calculation through the use of a continuum model whose band structure for small twist angles is captured with high accuracy for continuous twist angles. Here, we present a succinct introduction to the model Hamiltonian and the method used to obtain the linear response optical conductivity in twisted bilayer graphene for a twist angle that could support third-harmonic generation.

We set the lattice vectors of the monolayer graphene as ***a***_1_ = *a*_*G*_(1,0) and ${\boldsymbol{a}}_2 = a_G\left( {1/2,\sqrt 3 /2} \right)$, resulting in the first Brillouin zone consisting of a hexagon whose vertices are at a distance of *k*_*D*_ = 4π/3*a*_*G*_ from the Γ-point. The Hamiltonian of bilayer graphene with a relative twist angle *θ* in the continuum model for the K-valley is given by

1 $$H = \left( {\begin{array}{*{20}{c}} {h_b( + \theta /2)} & {T\left( r \right)} \\ {T\left( r \right)^\dagger } & {h_t( - \theta /2)} \end{array}} \right)$$

Here, *h*_*b*_ and *h*_*t*_ are 2 × 2 Dirac Hamiltonians rotated by ±*θ*/2, respectively, such that ${\it{h}}_{{\mathrm{b,t}}}\left( { \pm \theta /2} \right) = v_Fe^{ \pm i\theta \sigma _z/4}{\it{p}} \cdot \sigma e^{ \mp {\it{i}}\theta \sigma _{\it{z}}/{\mathrm{4}}}$, where the Fermi velocity $v_F = \sqrt 3 \left| {t_0} \right|a_G/2\hbar$, *t*_0_ = −3.1 eV^41^ and *σ*_*z*_ is the *z*-component of the Pauli matrix. *T*(***r***) denotes the interlayer coupling between two layers, which is given by

2 $$T\left( {\boldsymbol{r}} \right) = \mathop {\sum }\limits_{k = 0, \pm } e^{iQ_k \cdot r}T_{l,l^{\prime }}^k$$

where ***Q***_0_ = θ*k*_*D*_(0,−1), $${\boldsymbol{Q}}_ \pm = \theta k_D\left( { \pm \sqrt 3 /2,1/2} \right)$$, and

$${\it{T}}^0 = \left( {\begin{array}{*{20}{c}} \gamma^{\prime } & \gamma \\ \gamma & \gamma^{\prime } \end{array}} \right),{\it{T}}^ \pm = \left( {\begin{array}{*{20}{c}} \gamma^{\prime } & {\gamma {\it{e}}^{ \mp {\it{i}}2\pi /3}} \\ {\gamma {\it{e}}^{ \pm {\it{i}}2\pi /3}} & \gamma^{\prime } \end{array}} \right)$$

In the current paper, we take the lattice corrugation between the two layers into account by means of unequal intra- and intersublattice hopping terms between the layers, namely, *γ*′ = 0.10 eV and *γ* = 0.12 eV^44^.

According to linear response theory, the real part of the optical conductivity in the diagonal direction *σ*_xx_ up to linear order apart from the Drude weight located at *ω*=0 is given by ref. ^45^ as

3 $${\Bbb R}\left[ {\sigma _{xx}\left( \omega \right)} \right]/\sigma _0 = \frac{{16}}{\omega }{\int} {\frac{{d^2{\boldsymbol{k}}}}{{\left( {2\pi } \right)^2}}\mathop {\sum}\limits_{i,j} {\left[ {f\left( {{\it{\epsilon }}_{{\boldsymbol{k}},i}} \right) - f\left( {{\it{\epsilon }}_{{\boldsymbol{k}},j}} \right)} \right]} } |\left\langle {{\boldsymbol{k}},i|j_x|{\boldsymbol{k}},j} \right\rangle |^2\delta \left[ {\omega + ({\it{\epsilon }}_{{\boldsymbol{k}},j} - {\it{\epsilon }}_{{\boldsymbol{k}},i})/\hbar } \right]$$

in units of *σ*_0_ = *πe*^2^/2*h*, which is the optical conductivity of a graphene monolayer accounting for both spin and valley degeneracies. Here, *j*_α_ = −∂*H*/∂*k*_*α*_ is the current operator for the α = *x*, *y* directions, and $f\left( \epsilon \right)$ is the Fermi-Dirac distribution function.

The imaginary part of the optical conductivity in terms of its real part can be obtained by the Kramers–Kronig (KK) relation

$${\mathbb {I}}\left[ {{\upsigma }}_{x{\mathrm{x}}\left( {\upomega} \right)} \right] = \frac{2}{{\upomega }}{\cal{P}}{\int\nolimits_0^\infty} {d\xi \frac{{\xi ^2{\mathbb {R}}\left[ {{\upsigma }}_{{\mathrm{xx}}\left( \xi \right)} \right]}}{{{\upomega }}^2 - \xi ^2}}$$

Here, ${\cal{P}}$ is the Cauchy principal value to handle the improper integral.

We obtained the real and imaginary parts and the absolute value of the optical conductivity up to linear order in the diagonal direction for twisted bilayer graphene at a twist angle of 12.06° with respect to the electric field frequency, or transition energy ω in Fig. 3b.

We calculated the joint density of states based on the continuum model to investigate the dependency of possible light absorption on the twist angle as follows.

$$D_j\left( {E,\hbar {\upomega}} \right) = {\uprho}_V\left( {E - \hbar {\upomega}} \right)f_{FD}\left( {E - \hbar {\upomega}} \right){\uprho}_{\mathrm{C}}\left( E \right)\left[ {1 - f_{FD}\left( E \right)} \right]$$

where $\hbar {\upomega}$ is the photon energy and ${\uprho}_V\left( {E - \hbar {\upomega}} \right)$ and ρ_c_(*E*) are the number of accessible states for the upward transition in the valence (V) and conduction (C) bands, respectively. *f*_*FD*_ is the Fermi-Dirac distribution function, which is defined as $1/(e^{E - \hbar {\upomega} - E_f} + 1)$. Here, we set the Fermi energy *E*_*f*_ to be zero. Figure 3c shows the joint density of states for a transition energy of 2.35 ± 0.04 eV for different twist angles between 9.5° and 14°.

## Supplementary information

Supplementary Information for Enhanced third-harmonic generation by manipulating the twist angle of bilayer graphene
