# Supplementary material for: Enhanced third-harmonic generation by manipulating the twist angle of bilayer graphene
Source: Light Sci Appl. 2021 Jan 21;10:19. doi: 10.1038/s41377-020-00459-5 (PMC7820413; doi:10.1038/s41377-020-00459-5)
Supplement: Supplementary file 1 — Supplementary Information for Enhanced third-harmonic generation by manipulating the twist angle of bilayer graphene [file 41377_2020_459_MOESM1_ESM.docx]

Supplementary Information for

Enhanced third-harmonic generation by manipulating the twist angle of bilayer graphene

Seongju Ha^1^, Nam Hun Park^1,2^, Hyeonkyeong Kim^1^, Jiseon Shin^3^, Jungseok Choi^1^, Sungmin Park^1^, Ji-Yun Moon^1^, Kwanbyung Chae^1^, Jeil Jung^3,4^, Jae-Hyun Lee^1,5^, Youngdong Yoo^6^, Ji-Yong Park^1,7^, Kwang Jun Ahn^1^, and Dong-Il Yeom^1,7,^*

^1^Department of Energy Systems Research, Ajou University, 206 Worldcup-ro, Yeongtong-gu, Suwon, 16499, Republic of Korea

^2^Korea Research Institute of Standards and Science, Daejeon, 34113, Republic of Korea

^3^Department of Physics, University of Seoul, 163 Siripdaero, Dongdaemun-gu, Seoul, 02504, Republic of Korea

^4^Department of Smart Cities, University of Seoul, 163 Siripdaero, Dongdaemun-gu, Seoul, 02504, Republic of Korea

^5^Department of Materials Science and Engineering, Ajou University, 206 Worldcup-ro, Yeongtong-gu, Suwon, 16499, Republic of Korea

^6^Department of Chemistry, Ajou University, 206 Worldcup-ro, Yeongtong-gu, Suwon, 16499, Republic of Korea

^7^Department of Physics, Ajou University, 206 Worldcup-ro, Yeongtong-gu, Suwon, 16499, Republic of Korea

*Corresponding author: Dong-Il Yeom (e-mail: diyeom@ajou.ac.kr & Tel: +82-31-219-1937)

**
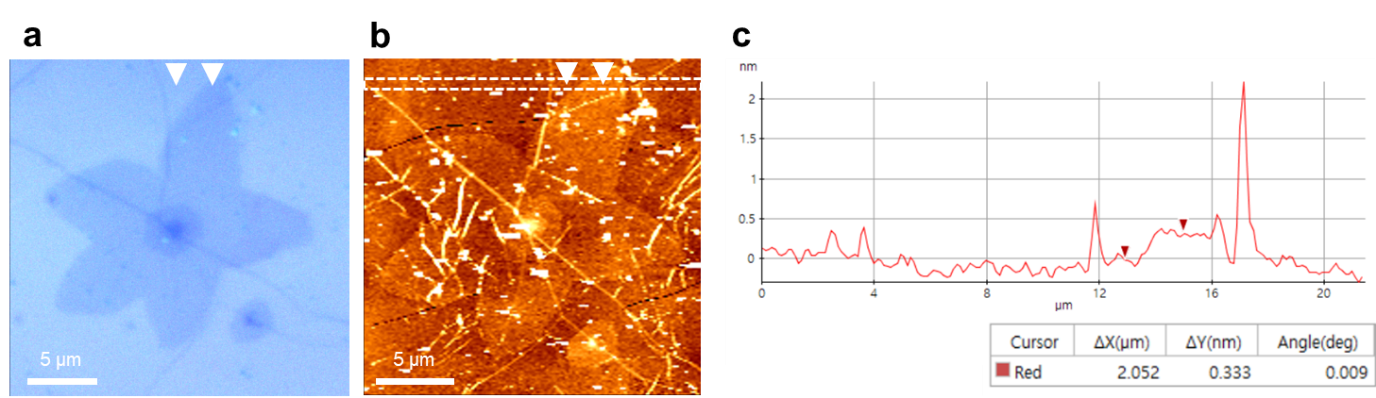
**

**Fig. S1 Surface and height profile of star-shaped graphene area examined by atomic force microscopy (AFM) measurement. a** Optical microscope (100×, 0.85 NA) image of an overgrown star-shaped graphene. **b** Surface profile of graphene in **a** by AFM measurement. **c** Averaged height profile of the white dashed square area in **b**. Red down-pointing triangles are corresponding to white down-pointing triangles in **a** and **b**, showing ~0.33 nm height difference from the monolayer graphene (MLG) region.

**
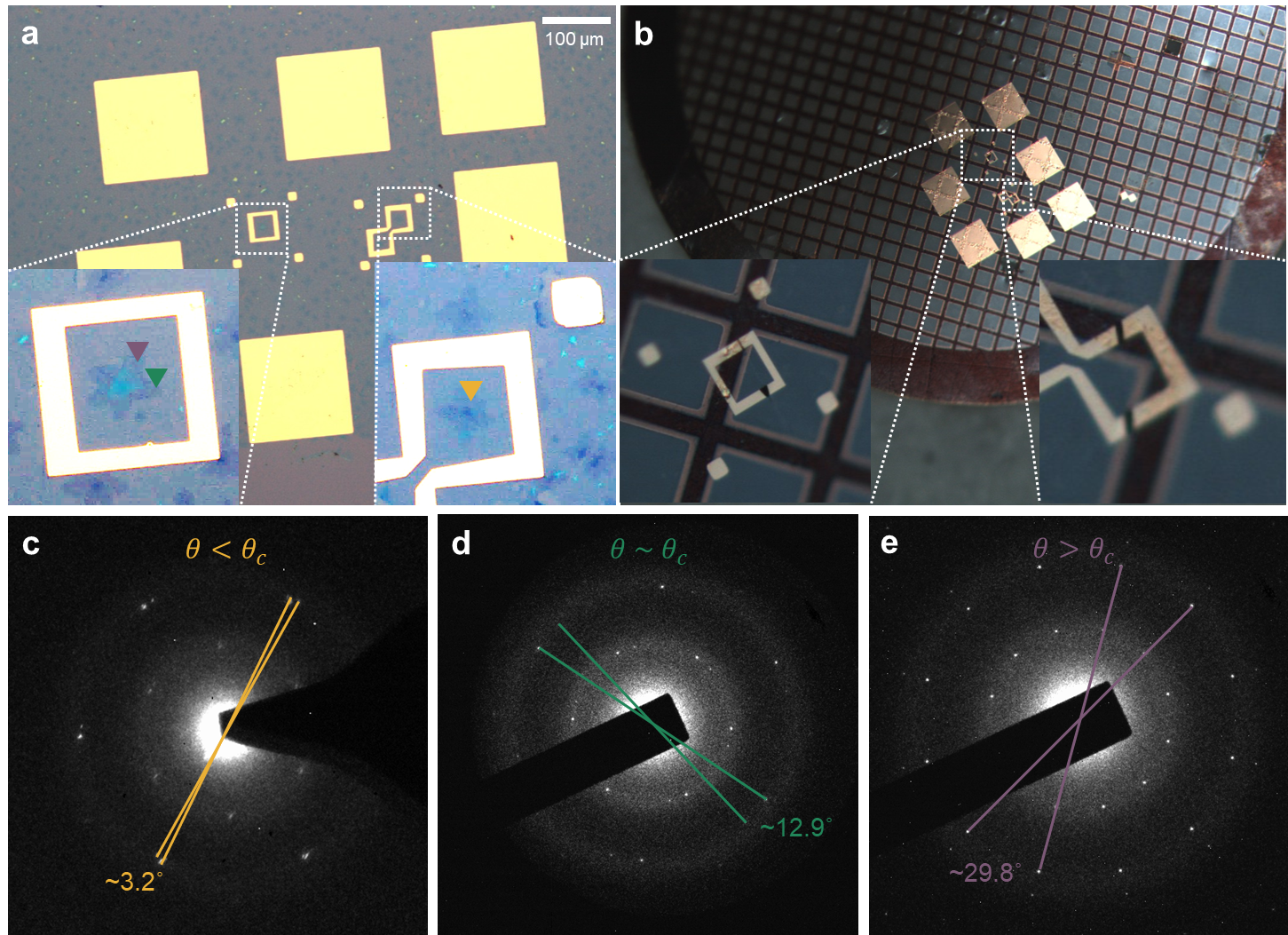
**

**Fig. S2 Selected-area electron diffraction (SAED) of tBLG grains. a, b** Optical microscope images of target tBLGs with Au markers before (**a**) and after (**b**) transferred onto TEM grid. **c-e** SAED images measured at tBLG grains with down-pointing triangles in **a**. The colors of triangles are corresponding with each tBLG grains; orange, green, and purple are for lower angle (~3.2°), near critical angle (~12.9°), and higher angle (~29.8°), respectively. For the SAED measurement, tBLGs were characterized by TEM (Thermo Fisher Scientific, Tecnai F30 operated at 300 kV for **c** and JEOL, JEM-2100F/HR operated at 200 kV for **d** and **e**).


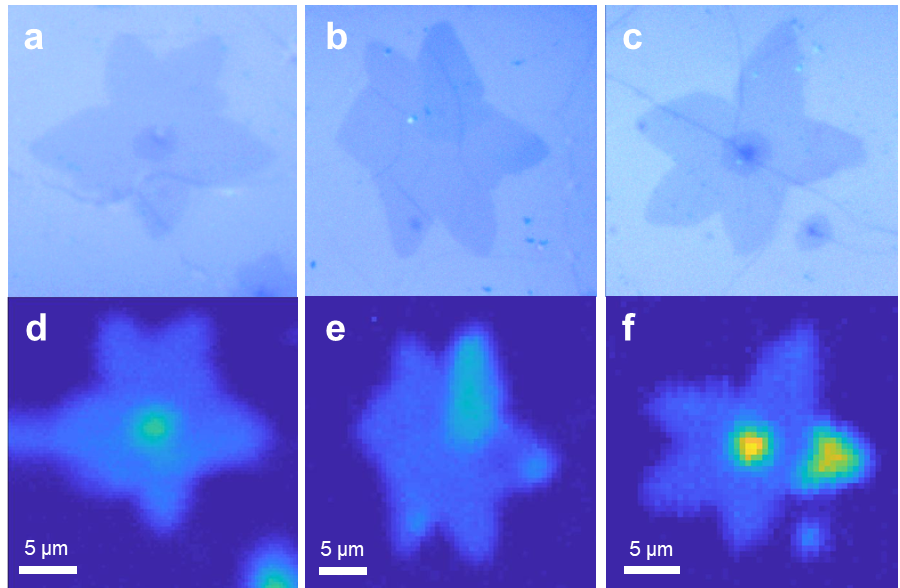


**Fig. S3 Third-harmonic generation (THG) in twisted bilayer graphene (tBLG) regions. a-c** Optical microscope (100×, 0.85 NA) images of various tBLG regions. **d-f** THG images corresponding to **a-c**, sequentially. To get THG images, tBLG regions were scanned by 1560 nm fs pump light with step of 0.3 μm in **d** and **e**, and 0.5 μm in **f**. Normalized THG intensities by that of MLG are 3.4 – 4.3 for **d** (indicating that all tBLG regions in **a** are far from the critical angle, $\theta_{c}$), 3.2 – 4.6 and 6.7 (upper region, slightly far from $\theta_{c}$) for **e**, and 3.3 – 3.8 and 11.2 (right side, near $\theta_{c}$) for **f**.


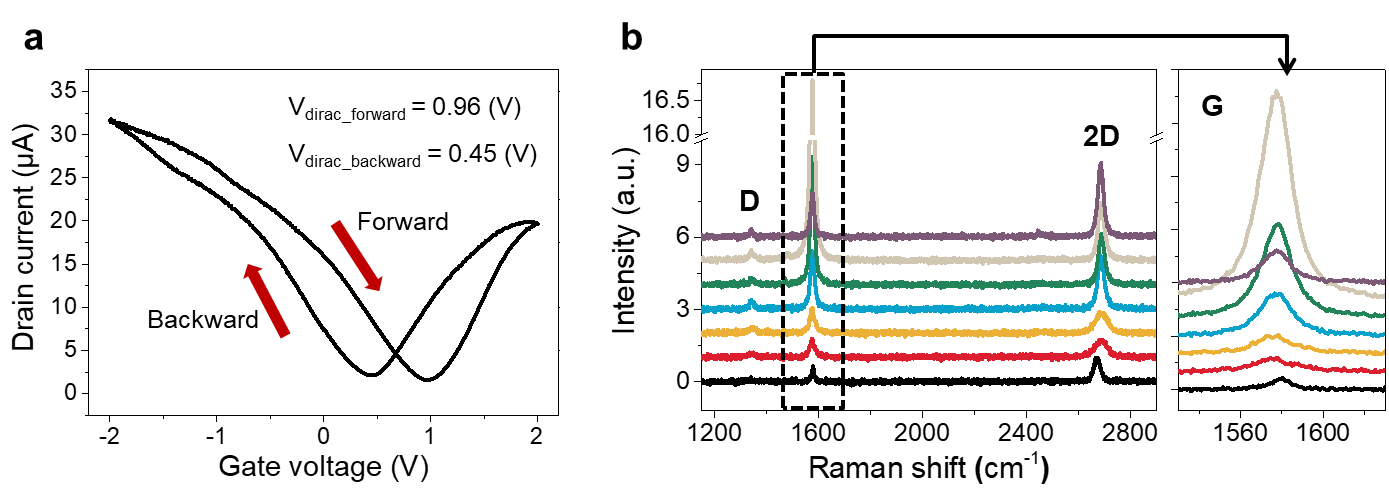


**Fig. S4 Electrical and optical characterization of top-gating tBLG device. a** Electrical transport properties of our graphene FET-like device with ion-gel gating. The sweep speed was 100 mV s^-1^, and the source-drain voltage was 5 mV. Both Dirac voltages for forward and backward direction measurements showed positive values, indicating that our CVD graphene was doped with p-type dopants possibly during the wet transfer process or by excessive charges at the interface of SiO_2_ and ion-gel coating, etc. **b** Raman spectra of tBLGs in the inset of main Fig. 4b. Gray- and green-colored tBLGs show resonantly increased Raman G peak intensities, where 2D/G ratio are 0.2 and 0.4, respectively.

**
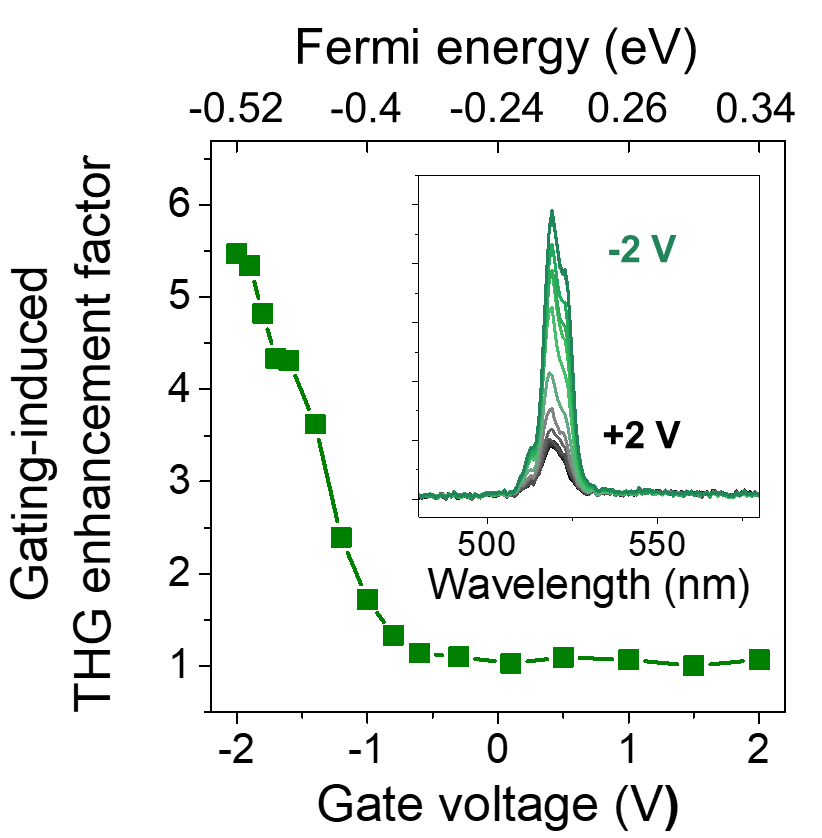
**

**Fig. S5 THG enhancement factor and spectral profile on gate voltages (**$\boldsymbol{V}_{\boldsymbol{g}}$**) in tBLG.** The measured THG in top-gated tBLG (the inset of main Fig. 4b) as a function of $V_{g}$ within the range from 2 V to –2 V. As tBLG became a p-doped state with the negative $V_{g}$, THG intensity increased as the previously reported behaviors in MLG^1,2^. The inset presents the gradual evolution of THG spectral profiles without a distortion along $V_{g}$. Fermi energy was calculated using an electric double layer capacitor (EDL) model in ref 3 using 1.21 μF cm^-2^ of electric double layer capacitance ($C_{EDL}$) and 0.705 V of Dirac voltage.

**
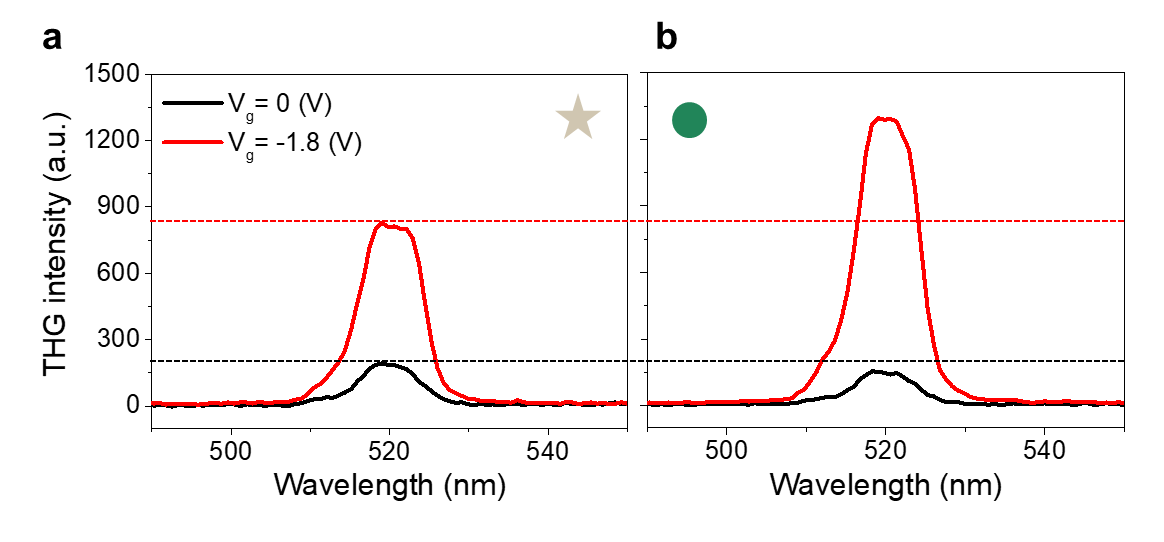
**

**Fig. S6 Representative THG spectra at** $V_{g}$ = **0 V and –1.8 V.** THG spectra from **a** Raman G-band resonant tBLG (grey-circled region in main Fig. 4b inset) and **b** tBLG with slightly lower twist angle than $\theta_{c}$ (green-circled region in main Fig. 4b inset). The initial THG intensity of G-band resonant tBLG was 1.25 times green-circled tBLG. But, electrically tuned THG intensity of green-circled tBLG showed the significant enhancement and became 1.7 times that of G-band resonant tBLG at $V_{g}$ = –1.8 V.


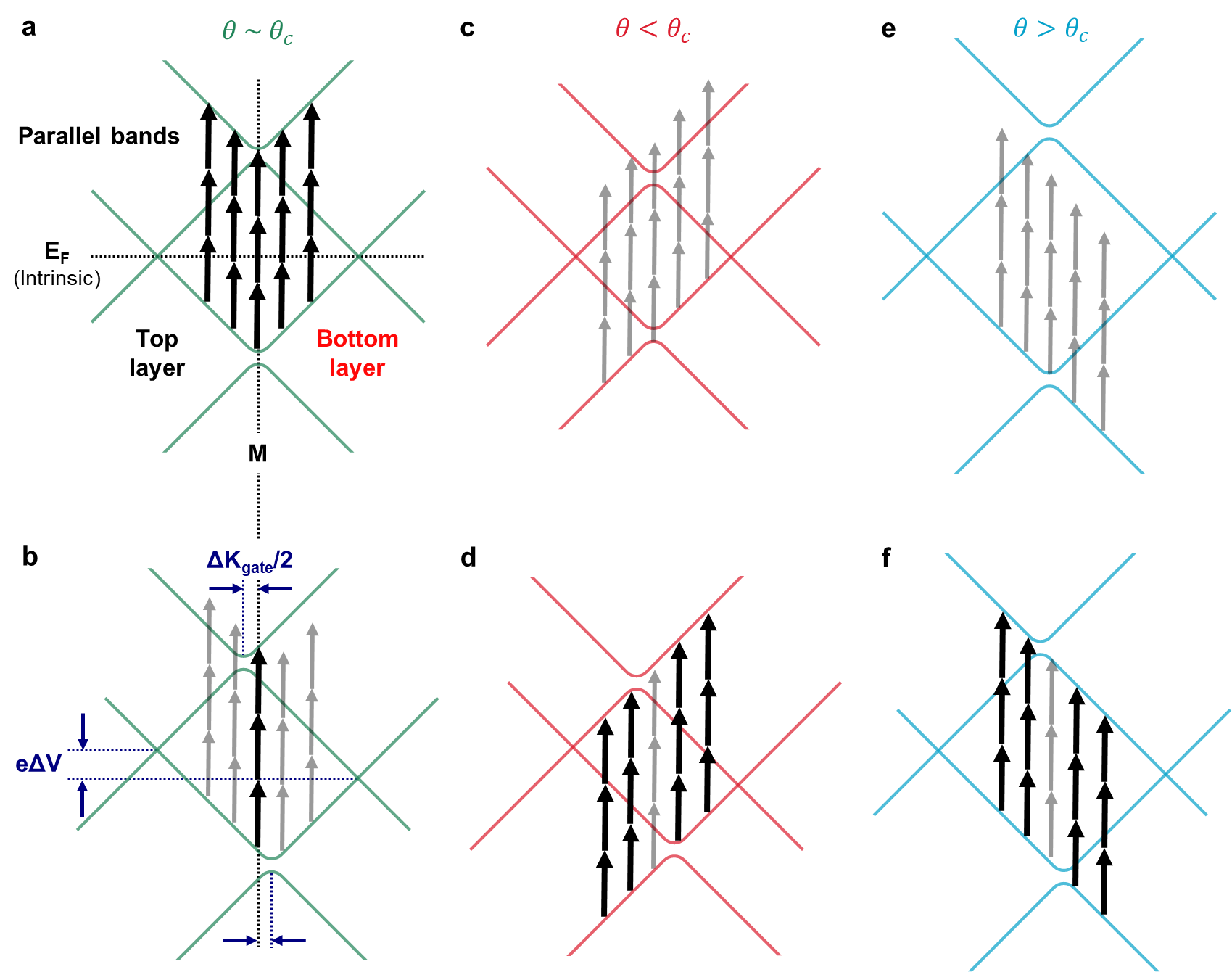


**Fig. S7 Schematics of asymmetric evolution in the electronic band structure and resonant three-photon transition (RTPT) state in tBLGs at an intrinsic and p-doped state.** Intrinsic electronic band structures and three-photon transition states of tBLGs with **a** $\theta_{c}$, **c** lower twist angle (${\theta< \theta}_{c}$), and **e** larger twist angle (${\theta> \theta}_{c}$). At this condition, the only tBLG with $\theta_{c}$ shows strong RTPT between parallel bands. However, when tBLG is in the p-doped state by electrical gating, RTPT can be reduced significantly due to the asymmetric evolution of electronic band structure as depicted in **b**. On the contrary, as shown in **d** and **f**, strong RTPT could be expected in tBLGs with ${\theta< \theta}_{c}$ or ${\theta> \theta}_{c}$ under specific electrical gating condition by parallel shift of electronic band structure with potential difference ($\Delta V$) between top and bottom layer graphene.

**References**

1. Jiang, T. et al. Gate-tunable third-order nonlinear optical response of massless Dirac fermions in graphene. *Nature Photonics* **12,**430–436 (2018).
2. Soavi, G. et al. Broadband, electrically tunable third-harmonic generation in graphene. *Nature Nanotechnology* **13,**583–588 (2018).
3. Park, N. H. et al. Strong electro-optic absorption spanning nearly two octaves in an all-fiber graphene device. *Nanophotonics* **9**, 4539-4544 (2020).
